# What’s missing in geographical parsing?

**DOI:** 10.1007/s10579-017-9385-8

**Published:** 2017-03-07

**Authors:** Milan Gritta, Mohammad Taher Pilehvar, Nut Limsopatham, Nigel Collier

**Affiliations:** Language Technology Lab (LTL), Department of Theoretical and Applied Linguistics (DTAL), University of Cambridge, 9 West Road, Cambridge, CB3 9DP UK

**Keywords:** Geoparsing, Geotagging, Geocoding, NER, NLP, NEL, NED

## Abstract

Geographical data can be obtained by converting place names from free-format text into geographical coordinates. The ability to geo-locate events in textual reports represents a valuable source of information in many real-world applications such as emergency responses, real-time social media geographical event analysis, understanding location instructions in auto-response systems and more. However, geoparsing is still widely regarded as a challenge because of domain language diversity, place name ambiguity, metonymic language and limited leveraging of context as we show in our analysis. Results to date, whilst promising, are on laboratory data and unlike in wider NLP are often not cross-compared. In this study, we evaluate and analyse the performance of a number of leading geoparsers on a number of corpora and highlight the challenges in detail. We also publish an automatically geotagged Wikipedia corpus to alleviate the dearth of (open source) corpora in this domain.

## Introduction

With the exponential increase in availability of public, free-format text generated through the accelerating use and adoption of internet-connected devices and the subsequent increase in social media usage, there is a greater opportunity for researchers and developers to utilise geographical information. Geographical data adds an additional dimension to the richness of data enabling us to know not just the what and when, but also the *where* of an event.

**Fig. 1:**
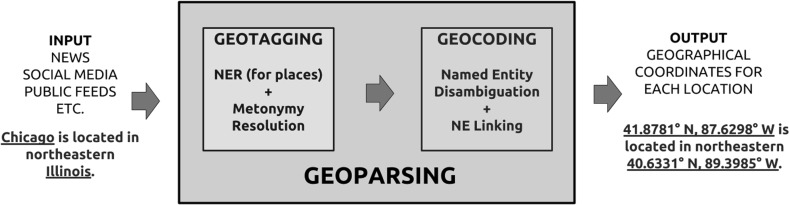
The geoparsing pipeline comprises two main stages geotagging and geocoding. Geotagging retrieves only literal toponyms (ideally filtering out metonymic occurrences) from text and generates candidate coordinates for each. Geocoding then leverages the surrounding context to choose the correct coordinate and link the mention to an entry in a geographical knowledge base

In geoparsing, the place names containing the geographical information are called *toponyms*, which must first be identified (called geotagging) and resolved to their geographical coordinates (called geocoding), see Fig. 1. This two stage approach is common in the domain and this is also how we evaluate the geoparsers. In an example sentence, “A publicity stunt in *Boston Common* causes surprise in *Washington, DC*”, the toponyms are “Boston Common” and “Washington, DC” since the action happens in both places.

Geotagging is a special case of Named Entity Recognition (NER), which is very much an open problem in NLP. The difference is, it only retrieves *locations* (no persons, organizations, etc.). Also, an important component of geotagging is Metonymy Resolution, which is discussed in Sect. 7.2.1. Geocoding, from an NLP point of view, is Named Entity Disambiguation (NED) followed by Named Entity Linking (NEL). Given a list of candidate coordinates for each location and the surrounding context, the goal is to select the correct coordinate i.e. disambiguate. Finally, each toponym is then linked to a record in a geographical knowledge base such as GeoNames.

This article provides the following two main contributions:

1. We provide a comprehensive survey and critical evaluation of state-of-the-art geoparsers with heterogeneous datasets. To the best of our knowledge, a detailed geoparsing survey such as this is currently unavailable.
2. We also present WikToR, a novel, large-scale, automatically generated and geographically annotated Wikipedia corpus for the purpose of alleviating the shortage of open source corpora in geoparsing research.

The rest of this paper is organized as follows. In Sect. 2, we provide the background to the geoparsing task, definitions and related research. In Sect. 3, we present the featured systems including related systems that did not qualify for our evaluation. In Sect. 4, we present our corpus (WikToR), its benefits and limitations, processing steps, quality checking and a sample article. In Sect. 5, we describe the metrics used, which is followed by result tables and their detailed interpretation in Sect. 6. We then discuss replicability and our experience in Sect. 7. Example errors committed by the surveyed systems are analysed in Sect. 7.2, which is followed by the conclusion.

## Background

As mentioned in the Introduction, geographical parsing is the task of identifying and resolving toponyms to their geographical coordinates. The exact definition of a toponym varies among researchers, lexicographers and developers. The most common (and ambiguous) dictionary definition is a *place name*. There are other suitable definitions for a toponym such as Wikipedia’s instructions for WikiProject contributors, which (as of June, 2016) contained the following:^1^ “In general, coordinates should be added to any article about a location, structure, or geographic feature that is more or less fixed in one place.” The United Nations Conference on the Standardization of Geographical Names^2^ define a toponym as *the general name for any place or geographical entity*. This is the best definition we found and we further add that a toponym is also *a name for a topographical feature*.

That means lakes, cities, monuments, colleges, and anything static (natural and artificial physical features of an area) are toponyms and require coordinates. In this paper, we only evaluate inhabited places (countries, cities, villages, regions, neighbourhoods, etc.), which is a subset of toponyms. This is the standard approach (DeLozier et al. 2015; Grover et al. 2010) in most geoparsing. However, other work (Gelernter and Balaji 2013) evaluated additional toponyms such as landmarks, buildings, street names, points of interest and outdoor areas (parks, etc). The datasets used in our evaluation contain many types of toponyms, although the most common types by far are inhabited places.

### Additional geotagging challenges

#### Metonymy

Toponyms should exclude metonymic readings i.e. comprise only locative interpretations (geographical territory, the land, the soil, the physical location). In a sentence “*London* voted to stay in the EU.”, London is a metonymic reading, substituting for the people of London, hence not a toponym. Another example is “*France* travelled to Spain for their friendly.”, which means the French team are heading out. A toponym has no capacity to act like a living entity (humans, organisations, etc.). To that end, any place name substituted in for the actual “actor” should not be considered a location.

In our work, however, we neither have the datasets to evaluate geoparsing performance for metonymic readings nor the geoparsers capable of this distinction. We further discuss the errors caused by the lack of understanding of metonymy in geoparsing in Sect. 7.2.1. There is no doubt that incorporating metonymy resolution into future geoparsing technology will increase performance (higher precision—fewer false positives).

#### Implementation choices

Another important factor that influences geotagging performance is the choice of software implementation with regards to NER. Most of the geoparsers tested do not implement their own NER, instead opting for open source software such as Apache NER^3^ or Stanford NER.^4^ Mota and Grishman (2008) tested the influence of the changing text time frame on the NER performance and found that over a period of 8 years, the performance of the NER tagger steadily declines, which was also the case in Lingad et al. (2013). Section 7.2 elaborates on the shortcomings of NER further.

### Additional geocoding challenges

In addition to the more manageable challenges such as misspellings and case sensitivity (analysed later), processing fictional and/or historical text presents an additional challenge for the geocoder (i.e. translating a location name such as “ancient city of *Troy*” into coordinates). Modern geographical place databases may not contain entries for historical places, many of which changed their names over time. The more prominent changes such as Tsaritsyn—(1925)—Stalingrad—(1961)—Volgograd or Vindobona—Vienna are covered however more obscure changes such as Aegyssus—(1506)—Tulcea (Romania) are not included. This problem can be solved by using Pleiades^5^ (Simon et al. 2015), a community-built gazetteer of ancient places. At the time of this publication, it contained  35,000 ancient places. Named Entity Linking algorithms such as REDEN (Brando et al. 2016) can help with selecting the right candidate from a list of potential entities.

Errors can also occur with places which have historically changed names, such as Belfast, Australia, which became Port Fairy or when Limestone Station, Australia became Ipswich. The harder problem, however, is geocoding fictional places (islands, towns, states, etc.) such as Calisota (California + Minnesota) from Walt Disney Comic Books. The same applies to virtual places like Zion from the film Matrix or other fictional locations. These occurrences, however, should be infrequent in modern popular discourse.

## Methodology

### Selecting systems

The comparison we conducted here was not restricted to only academic (accompanied by a scientific paper) parsers. In order to produce the broadest survey possible, we included commercial, open-source and academic solutions and used the following selection criteria:

1. The paper (academic geoparsers only) describing the methodology was published in 2010 or later
2. The geoparser is either publicly available (through APIs) or downloadable (as source code/binaries)
3. The geoparser performance (reported in a paper or trialled through a demo version) was near the state-of-the-art.

There are several related NER/NED tools such as OpeNER,^6^ Thomson Reuters Open Calais,^7^ however, these do not disambiguate down to the coordinates level, hence do not qualify as geoparsers. AIDA^8^ (Yosef et al. 2011) is another excellent NED tool for named entity identification and disambiguation, however similarly to OpeNER, the output is a link to a Wikipedia/DBpedia page, which may or may not contain coordinates. Despite their usefulness, they do not qualify as full geoparsers, hence do not feature in this survey. Finally, we were not able to obtain a working version of the Geolocator by Gelernter and Balaji (2013), Zhang and Gelernter (2014).

### The featured geoparsers

*Cartographic Location And Vicinity INdexer* 9 (CLAVIN) is an open-source geoparser that employs context-based geographic entity resolution. It was downloaded from GitHub on 5 December 2015. CLAVIN employs fuzzy search to handle incorrectly-spelled location names. It also recognises alternative names and utilises other open-source technologies such as Apache OpenNLP^10^ as well as intelligent heuristics-based combinatorial optimisation to identify precisely which toponym sense was referred to in the text. The data for CLAVIN comes from the GeoNames Knowledge Base.^11^ As far as we can tell, there is no academic publication available for this open source project. Additional information can be found on the project website.^12^

*Yahoo!PlaceSpotter* 13 is a commercial geoparser that identifies places in unstructured text such as web pages, RSS feeds, news or just plain text files, all with multilingual support. Yahoo!’s proprietary Geo-informatics database information consists of six million (and growing) named places including administrative areas, settlements, postal codes, points of interest, colloquial regions, islands, etc. PlaceSpotter also delivers bounding boxes and centroids of named places. The API was used from January through March 2016 to process the featured corpora. PlaceSpotter identifies locations using WOEID (Where on Earth ID), which always reference place concepts. For example, “New York”, “New York City”, “NYC”, and “the Big Apple” are all variant names for WOEID 2459115. The geoparser also has an understanding of geographical focus of the document i.e. where is this document talking about, which may be useful for many applications.

*The Edinburgh Parser* (Grover et al. 2010; Tobin et al. 2010) is an end-to-end system, developed in-house and partitioned into two subsystems, a geotagger and a geocoder. The geotagger is a multistep rule-based NER which makes use of lists of place names and person names for personal and location entity recognition. The gazetteers, Unlock^14^ (decommissioned in 2016) and GeoNames, which can be chosen at run-time, provide the location candidates together with metadata such as population, coordinates, type, country, etc.

The rule-based geocoder then uses a number of heuristics such as population count, clustering (spatial minimization), type and country and some contextual information (containment, proximity, locality, clustering) to score and rank the candidates and finally choose the correct one. If there is no entry in the gazetteer, the place remains unresolved. As with every other tested geoparser, the evaluation was performed end-to-end (pipeline) to simulate real world usage. The binaries for the geoparser were downloaded^15^ on 21 Decmber 2015.

*Topocluster* (DeLozier et al. 2015) models the geographic distribution of words over the earth’s surface. The intuition is that many words are strong indicators of a location thus good predictors given contextual cues. The geotagging is done using standard Stanford NER. The geocoding is performed by overlaying the geographic clusters for all words in the context (up to 15 each side) and selecting the strongest overlapping point in the distribution. Topocluster uses a geographically tagged subset of Wikipedia to learn this spatial distribution of word clusters i.e. it is trained to associate words in the vocabulary with particular coordinates. In addition to the single point in the world, a gazetteer entry closest to the predicted coordinates is chosen as the final location candidate.

Although Topocluster can work without a knowledge base, resolving toponyms to a single pair of coordinates, for best performance, it does require a gazetteer (mainly GeoNames plus a small Natural Data^16^ dataset of just 500 referents). Domain optimisation to each dataset is required for best results (in our evaluation, we do not adapt to new domains for a fair comparison with other systems, which also use default setups). The experiments outlined in their publication have shown that the domain parameters are volatile and corpus-specific, which may adversely affect performance in the absence of corpus adaptation. The final version of the software was downloaded from GitHub on the 18 December 2015.

*GeoTxt* 17 (Karimzadeh et al. 2013) is a web-based geoparser specialising in extraction, disambiguation, and geolocation of toponyms in unstructured micro-text, such as Twitter messages (max 3900 characters, approx 750 words). Identifying and geographically resolving toponyms in micro-text is more challenging than a longer article such as a news report due to fewer contextual features. Much like the previous systems, it works in two steps, geotagging (extracting toponyms) using three NER taggers, Stanford NER,^18^ ANNIE,^19^ Illinois NER^20^ and geocoding.

Geocoding (disambiguation) is performed using the GeoNames Web Service to generate a list of candidates. To rank and score them, they use: geographic level, e.g. country, province or city of the place name in text (when provided), Levenshtein Distance between the candidate name and the one mentioned in text and population with higher priority given to places with higher population. GeoTxt also uses spatial logic to leverage other place name mentions in context such as “I love *London*, I love Canada!”. This will resolve to London, Ontario, Canada. The parser was evaluated from February to March 2016.

## Corpora

There exists a challenge that all researchers in this field currently face and it is the lack of freely available geotagged datasets. We are aware of a few datasets such as the free and open War Of The Rebellion^21^ by DeLozier et al. (2016), which was released at the ACL 2016 conference. A geotagged Twitter corpus was announced by Wallgrün et al. (2014), which should help alleviate the problem. Another geotagged dataset called TR-CONLL by Leidner (2006) exists although the fees, terms and conditions of licensing are unclear. The corpus relies on Reuters data (a subset of the CONLL Shared Task 2003 dataset), which is not publicly available thus not conducive to open research. Finally, the ACE 2005 English SpatialML is a broadcast news and broadcast conversation geotagged corpus, also suitable for geoparsing evaluation. However, it is only available for a fee,^22^ which once again does not support open research and replication.

### Our corpus

As part of our contribution, we introduce a large, programmatically created corpus called WikToR (Wikipedia Toponym Retrieval) to allow for a comprehensive and reliable evaluation on a large corpus. The Python programming script^23^ uses three sources of data to construct WikToR, the GeoNames^24^ database dump, the GeoNames Wikipedia API^25^ and the Wikipedia API^26^ using a Python wrapper^27^ (Accessed: March 2016).

**Fig. 2:**
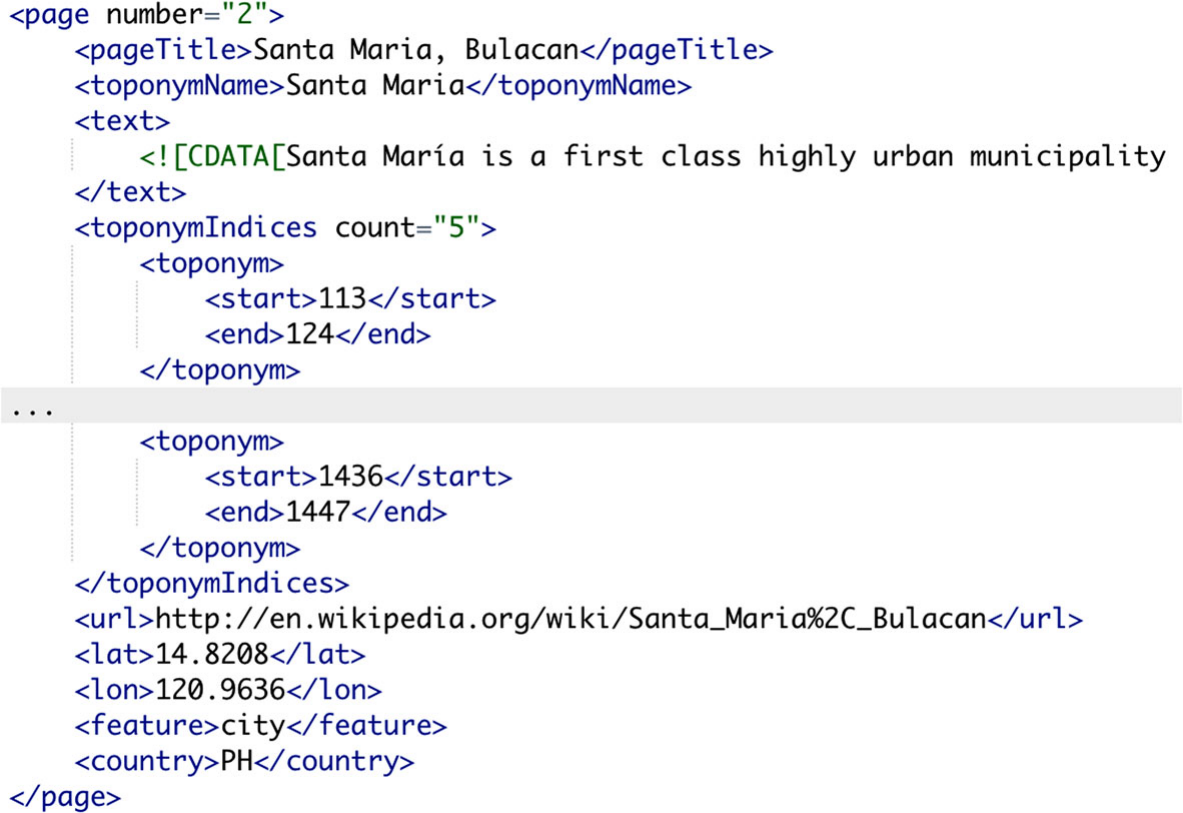
A sample article from WikToR. The article has been shortened

#### Processing steps

Starting with approximately 10 M entries in the GeoNames database, the following steps were used to create the final corpus:

1. Locations with the same name had to be separated by a large (1000 km) distance to be distinct and to remove duplicates.
2. The most ambiguous locations (ones with the highest count in step 1) were processed first. For instance, the five most ambiguous GeoNames locations are Santa Maria (26 entries), Santa Cruz (25), Victoria (23), Lima (19) and Santa Barbara (19), although Wikipedia does not have a page for each.
3. For each location, the Wikipedia pages were downloaded using GeoNames API.^28^
4. Each page had to be tagged with one of [adm1st, adm2nd, adm3rd, city, country, isle] feature. codes^29^ to be accepted.
5. The final article is a Wikipedia page with the first paragraph(s) that exceed 200 word tokens of context (see Fig. 2).

#### Benefits and limitations

The benefits:

Because WikToR has been created programmatically, it delivers great consistency of annotation (see Sect. 4.1.3 on Quality Checking). The corpus can be updated and extended easily as the code^30^ used to generate it was published alongside the corpus. The corpus presents a hard challenge, particularly for the geocoder. The 5000 unique Wiki pages contain some relatively obscure locations from around the world, at least 1000 km apart so a mistake is costly.

**Fig. 3:**
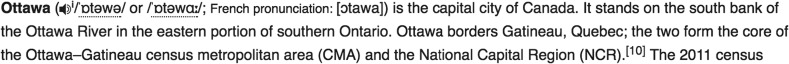
An article from Wikipedia about Ottawa. All necessary disambiguation information is provided in the first sentence of each article, which is included in every WikToR sample

Locations are deliberately highly ambiguous in that they share their name with other namesake locations for instance Lima, *Peru*, Lima, *Ohio*, Lima, *Oklahoma*, Lima, *New York* and so on. Finally, all articles are resolvable (distinguishable) since they contain the lead section of each Wikipedia article^31^ providing enough disambiguation to precisely and confidently resolve each annotated location, see Fig. 3.

The limitations:

The “one sense per discourse” (Gale et al. 1992) will not hold if processing the whole corpus at once (5000 articles include many namesake places). We recommend processing one article at a time. Secondly, for geotagging, the corpus can only be used to evaluate the accuracy (rather than F-Score) since not all locations were annotated. For example, a page about “Victoria” will only be annotated with the occurrences of “Victoria”, hence no precision or recall. This does not affect the geocoding metrics.

#### Quality checking

The advantage of a programmatically created corpus is the consistency of annotation. The corpus and the Python code that were used to create it is available for download from GitHub. In terms of data integrity, we rely on GeoNames and Wikipedia for the correctness of the coordinates, titles, URLs, feature and country codes.

All page text was retrieved using Wikipedia API for Python,^32^ which is an open source library for accessing Wikipedia. The indexed toponym occurrences in pages include only whole location words i.e. “Brazil” NOT “Brazilian”, all of which were unit-tested for their correctness. This includes checking for the presence and correct format of the page title, article numbering, text length, coordinates, URL, toponym indices, country code and toponym type.

In the light of the licensing and availability conditions, we chose to benchmark the 5 systems on 2 available corpora (WikToR and LGL). Each parser uses only one best setting to parse all corpora. The datasets we used in our experiments were:

1. Local Global Corpus (LGL)—The corpus contains a set of 588 news articles, collected from smaller, geographically-distributed newspapers. Each article’s toponyms were manually tagged and resolved. None of the toponyms in the corpus are larger than a US state (18% are US state or equivalent). The most popular countries in the corpus were USA (3252 locations), Russia (166), UK (140), Canada (129), Georgia (119), Israel (84), Palestine (84), Egypt (72), Sudan (49), Lebanon (33). Around 16% of toponyms in LGL do not have coordinates so we decided not to use those in order to evaluate all systems end-to-end. For a more detailed description, see Lieberman et al. (2010).
2. Wikipedia Toponym Retrieval (WikToR)—is an automatically created and geotagged corpus (full description in the next section). Corpus statistics: 5000 geographically unique Wiki pages, 1906 of those lexically unique. Pages are ambiguous such as Lima, *Peru*, Lima, *Ohio*, Lima, *Oklahoma*, Lima, *New York* (2.6 pages per location name on average, median = 1). The average location occurrences per Wiki page is 8.5 (median 7) and the average page word count is 374 (median 302). Finally, all toponyms in WikToR have coordinates.

## Evaluation

### Unsuitable metrics

There is currently no clear agreement on which metrics are best, for instance: *precision@k* (for geotagging) asks: Is the correct result in the first **k** predicted answers? However nearby answers are treated as equally correct as distant answers (and when k = 1, it’s equivalent to standard precision); *accuracy@k km/miles* (geocoding) is a better metric however it does not distinguish between similar predictions, for instance, 5 km away and 50 km away are equally correct when k = 50. This will hide differences in error distribution.

**Fig. 4:**
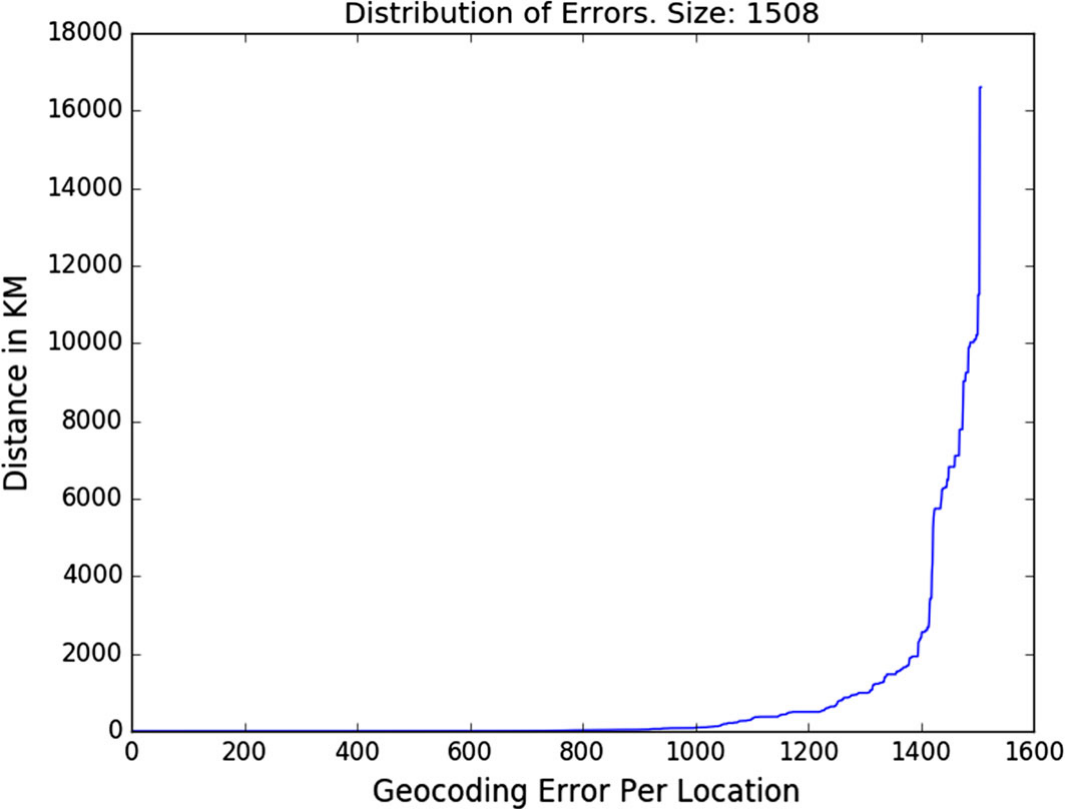
A typical power law distribution of geocoding errors in KMs away from the gold location. Most errors are *relatively* low but increase very fast for the approximately last 20% of locations. This is a typical pattern observed across different systems and datasets

The *mean error* (geocoding) is too sensitive to outliers plus normal distribution of error distances should not be presumed (as this tends to follow the power law, see Fig. 4) This means that a handful of very large errors has the potential to make the average error go up noticeably thus unfairly punishing a geoparser. In this paper, we only show mean error and accuracy@k for backward compatibility and comparison with earlier work.

### Selected metrics

We evaluated geoparsing as a pipeline (Input $\rightarrow$ Geoparser (as a black box) $\rightarrow$ Output), then scored each stage separately. The geotagging performance is measured using the *F-Score* for the LGL corpus, *Accuracy* for WikToR (see corpus limitations in Sect. 4.1.2). The geocoding performance is measured using the *AUC* and *Median Error* for both corpora.

**Fig. 5:**
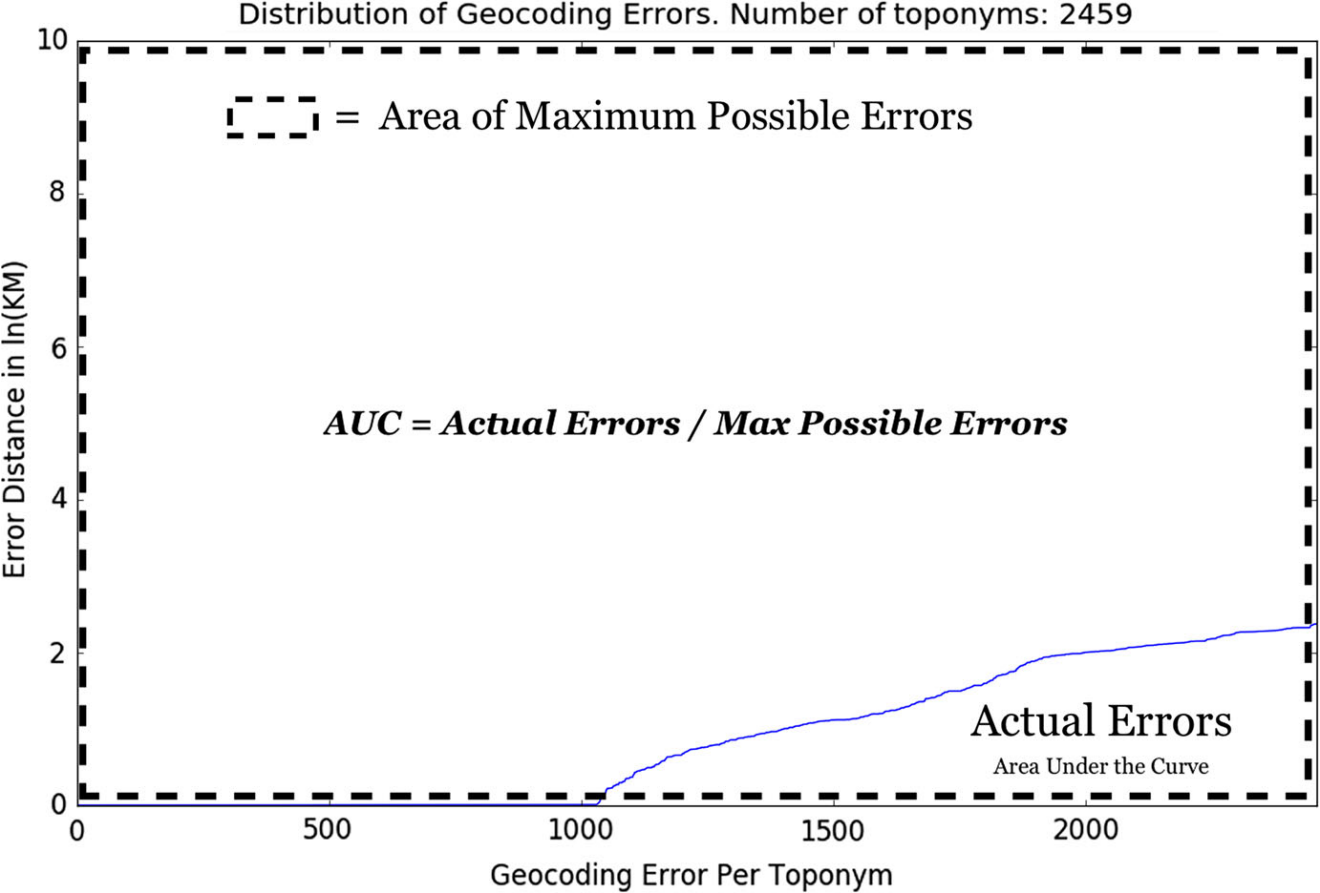
How to calculate the AUC, a visual illustration. Lower scores are better. Figure 4 shows the same error data in its original form (before applying the natural logarithm)

*Area Under the Curve* (AUC)—To the best of our knowledge (Jurgens et al. 2015) first introduced the AUC for geocoding, which is a type of extension of the accuracy@k km/miles. AUC makes a fine-grained distinction between the individual errors rather than just a simple binary evaluation as with accuracy@k (either within k km/miles or not). This is accomplished by quantifying the errors as the area under the curve (see Fig. 5). The smaller the area under the curve, the more accurate the geocoder.

*AUC* has a range of 0–1 since it assumes that an error cannot be greater than the furthest distance between two points on earth (20,038 km). By taking the natural logarithm of the error distance, AUC ensures that the difference between two small errors (say 10 and 50 km) is more significant than the same difference between two large errors (say 1000 and 1040 km). AUC is superior to the popular accuracy@k because it computes all errors giving a true like-for-like comparison between geoparsers.

*Precision* is the proportion of entities correctly identified as locations (true positives). In cases where non-location entities were identified as locations, these cases count as false positives. The formula for calculating precision is: true positives/(true positives + false positives) (range: 0–1).

*Recall* is the proportion of all locations identified (true positives). In cases where annotated locations were not identified as such, these count as false negatives. The formula for calculating recall is: true positives/(true positives + false negatives) (range: 0–1).

*F-score* is the harmonic mean of precision and recall and is calculated as (2 * Precision * Recall) / (Precision + Recall).

*Accuracy*—Accuracy measures the percentage of correct predictions out of a total of expected predictions (ranging from 0 to 1). In the case of WikToR, the accuracy measures the percentage of correctly identified locations by the system out of the total number of gold annotated locations.

*Median (natural log) Error Distance*—The error distance is a distance from the actual (gold) location (latitude, longitude) to the location (latitude, longitude) predicted by the geocoder. A median error is more informative than a mean error, which only has relevance if error distances are normally distributed (having plotted the error distributions, they follow the power law).

*Accuracy@161km (geocoding only)*—For backwards compatibility with previous work on geocoding, we also show accuracy@161 km for the geoparsers. This metric shows the percentage of geocoding predictions, which were no more than 161 km away from their true coordinates. Lower numbers are more desirable. The empirical reason for the choice of 161 km as the cut-off distance is not clear from related work (Cheng et al. 2010).

### Running the geoparsers

During evaluation, each geoparser was treated as a black box, an end-to-end system receiving a single news article (LGL) or a single Wikipedia article (WikToR), then evaluating separately. NB: Most systems do not allow for geotagging, geocoding to be run individually, hence the “black box”. Lastly, the Edinburgh Parser is the only system that allows toponyms to have NIL referents i.e. no coordinates assigned. This only happens in 2–4% of the cases. We decided to remove these from evaluation for a fairer comparison.

## Results

### Scoring in geotagging

*Exact and Inexact*—Because of subtle differences between test file annotation and real-world geoparsing, we used two types of scoring for the geotagging stage. Exact matching compares the output and annotation exactly. For example, if “Helmand” is annotated, but the output is “Helmand Province”, this is both a false positive and a false negative. Same applies for“Birmingham” and “District of Birmingham”. Inexact matching for these examples is more lenient and accepts both answers, resulting in a true positive i.e. no errors.

### Geotagging results

Table 1 shows the geotagging performance on LGL, and Table 2 on WikToR (accuracy only, see Sect. 4.1). For geotagging, we report two scores, the exact i.e. only exact text span matches and inexact (in brackets) i.e. “Avoyelles Parish County” and “Avoyelles Parish” are both correct.

**Table 1:** Geotagging performance on LGL

LGL	Precision	Recall	F-score
GeoTxt	0.80	0.59	0.68 (*0.74*)
Edinburgh	0.71	0.55	0.62 (*0.67*)
Yahoo!	0.64	0.55	0.59 (*0.67*)
CLAVIN	**0.81**	0.44	0.57 (*0.59*)
**Topocluster**	**0.81**	**0.64**	**0.71** (**)

The bold values indicate the best performance for that metric out of all tested systems

Numbers in brackets are improved scores for inexact matches such as geotagging “Helmand” instead of “Helmand Province” or vice versa

** Inexact scores not available due to the system’s non-standard output

Even with inexact matching, no geoparser crosses the F-Score=0.8 threshold. Topocluster and GeoTxt, both of which use Stanford NER, performed almost identically on LGL (0.68 and 0.71 F-Score) although less well on WikToR (0.51 and 0.54 accuracy). The rest of the geoparsers use a combination of techniques for NER, open-source, proprietary and rule-based. The rule-based Edinburgh geoparser performed best on WikToR (3rd best on LGL) while the rest faltered, particularly CLAVIN (only 0.21 accuracy).

In the Introduction, we asserted that NER is very much an open NLP task, particularly for place names. The three geoparsers that integrate external NER software (GeoTxt, Topocluster, CLAVIN), leave a lot of room for improvement as shown in the tables. Wikipedia text proved to be the greater challenge, a finding mirrored by Balasuriya et al. (2009). The ultimate goal for geotagging performance should be F-Scores of 0.9+ or alternatively Fleiss’ kappa (Fleiss and Cohen 1973) of 0.81+ in multiple domains, which is approximately human level performance. Until such time, NER, which is a fundamental part of the geographical parsing task, remains an unsolved problem (Marrero et al. 2013) in NLP.

**Table 2:** Geotagging performance on WikToR

WikToR	Accuracy	Accuracy (inexact)
GeoTxt	0.51	*0.51*
**Edinburgh**	**0.65**	***0.66***
Yahoo!	0.4	*0.5*
CLAVIN	0.21	*0.22*
Topocluster	0.54	(**)

The bold values indicate the best performance for that metric out of all tested systems

Inexact example: geotagging the “City of London” instead of only “London” and vice versa

** Not available due to the system’s nonstandard output

From a parsing speed point of view, we estimated the fastest geoparser (CLAVIN—processing WikToR in ~2 min) to be around 10,000 times faster than the slowest (Topocluster—processing WikToR in 2 weeks and 8 h). However, the speed advantage did not translate into a geotagging advantage. The Edinburgh geoparser did best overall, its processing speed was more than adequate.

### Geocoding results

Table 3 shows the geocoding results for LGL and Table 4 for WikToR. All geoparsers (except Yahoo!—proprietary) use the same knowledge base (GeoNames) for a fair comparison of results. The AUC**E** metric is the same as AUC, but this time, run on an identical set of toponyms (i.e recognised by all five geoparsers, 787 for the LGL dataset and 2202 for the WikToR dataset). In the equal comparison, the AUC scores improve significantly (most likely due to the selection bias), however, the order of the geoparsers is mostly unchanged.

**Table 3:** Geocoding results on LGL

LGL	AUC	Med	Mean	AUCE	A@161
GeoTxt	0.29	0.05	2.9	0.21	0.68
**Edinburgh**	**0.25**	1.10	**2.5**	0.22	**0.76**
Yahoo!	0.34	3.20	3.3	0.35	0.72
CLAVIN	0.26	**0.01**	**2.5**	**0.20**	0.71
Topocluster	0.38	3.20	3.8	0.36	0.63

The bold values indicate the best performance for that metric out of all tested systems

Lowest scores are best (except A@161). All figures are exponential (base **e**) (except A@161), so differences between geoparsers grow rapidly

**Table 4:** Geocoding results for WikToR

WikToR	AUC	Med	Mean	AUCE	A@161
GeoTxt	0.7	7.9	6.9	0.71	0.18
Edinburgh	0.53	6.4	5.3	0.58	0.42
**Yahoo!**	**0**.**44**	**3.9**	**4.3**	**0.53**	**0.52**
CLAVIN	0.7	7.8	6.9	0.69	0.16
Topocluster	0.63	7.3	6.2	0.66	0.26

The bold values indicate the best performance for that metric out of all tested systems

Lowest scores are best (except A@161). All figures are exponential (base **e**) (except A@161), so differences between geoparsers grow fast

Our intuition that WikToR is a hard geocoding challenge was confirmed in the poor geocoding scores (N.B. scores are exponential, base **e**). There was a role reversal, CLAVIN (last in geotagging) excelled in this sub-task while Topocluster (first in geotagging) came last. This illustrates an important point, namely the integrity and performance of the entire pipeline. It is not sufficient to excel only at speed (CLAVIN) or geotagging accuracy (Topocluster) or geocoding performance (Yahoo!). A great geoparser must do all three well and the only one to manage that is the Edinburgh Parser. We also emphasise that there is much room for improvement in this technology, see Sect. 7.2.

The geocoding task revealed a difference between the two corpora. For LGL, which is a news-based corpus, AUCE scores decreased. For WikToR, which is a Wikipedia-based corpus, AUCE scores increased. We saw easily detectable toponyms in LGL resulting in easier geocoding, however, easily detectable toponyms in WikToR resulted in harder geocoding. This demonstrates the ambiguity of WikToR, which we asserted in Sect. 4.1. The corpus was built with location ambiguity as a goal so that prominent locations around the world have many namesakes, and are thus harder to geocode.

With LGL, we hypothesise that prominent locations tend to be the most frequent “senses” of the toponym i.e. “Lima” most likely refers to “Lima, Peru” rather than “Lima, New York”. In contrast, WikToR has seven unique entries for “Lima”, which is a prominent geographical entity and should be easy to detect. However, resolving “Lima” in WikToR is not a straightforward assignment of the most well-known location entity by that name as seems to be the case in LGL. This makes WikToR the suitable benchmark for the ultimate geocoding challenge on which all other geoparsers should be tested to evaluate how well they can deal with high ambiguity.

Lastly, with respect to fairness of comparison, we briefly address Yahoo! Placemaker’s proprietary database, which is the only one not to use GeoNames. It’s not possible to empirically measure the differences between private knowledge bases, however, we think this will not unduly affect the results for the following reasons. The number of records in the databases is comparable, around 6 M for Yahoo! and 11 M for GeoNames, which is easily within an order of magnitude. Secondly, we do not expect the coordinates for any particular location to differ markedly between the knowledge bases. Even a difference of up to two-digit kilometers (which is still adequate) will not significantly alter the final AUC scores. Finally, 82% of LGL and 95% of WikToR locations are no larger than a US state or equivalent, of which most are cities and counties. It is the implementation of the geocoding algorithm that is the greatest determiner of final geocoding performance.

### How to improve evaluation further

In future work, evaluation of precision can be further improved by taking the magnitude and type of geocoding errors into consideration. The error should be adjusted based on the size of the area of the location (geographical granularity). For example, a 100 km error for a small town (50 sq km) is hugely costly compared to a 100 km error for a large country (650,000 sq km). The larger the entity, the more disagreement exists as to its exact geographical centre. This can be mitigated by evaluation becoming more forgiving as the area of the location entity increases. Alternatively, one can stratify the evaluation and present results at each level.

In current approaches, all geocoding errors are treated equally. Our suggested new kind of error contextualization befits the intuition that tolerance for a geocoding error should grow with the size of the location entity. An example application can utilise the GeoNames (premium subscription^33^) database to retrieve the polygon coordinates for the calculation of the area of the location entity. Then apply a linear scaling factor such as a cube root to calculate an error tolerance. For example, the area of Greater London is $\sim$1500 sq km so $\root 3 \of {1500} = 11.45$ meaning an error of 11.45 km can be considered a “calibration” error for the size of Greater London. Similarly, for France ($\sim$644 K sq km), the error tolerance would be $\root 3 \of {644000} = 86.4\,\hbox {km}$.

## Discussion

### Replicability

Verifiability and disproof are the cornerstones of the scientific method. In the wider scientific community, it is commonly known that many studies are not replicable (Peng 2011; Leek and Peng 2015) and this is also the case with *irreproducible software* (Sufi et al. 2014). We would like to add value by sharing some key observations from our experience during the making of this publication. It is important that a detailed methodology and/or software be published with the scientific paper (including any data) (Gentleman and Lang 2012).

This issue repeatedly arose in our experimentation. Some systems did not have a working version of the parser obtainable from the official source. Some of these took many weeks of time and/or a frequent email exchange to rectify in order to enable reproduction of the reported results. In other cases, the waiting time for essential software was around a month only after a written request was made. Another software was prohibitively cumbersome to set up, which does not aid replication. On the positive, some parsers were readily runnable with little or no setting up and helpful cooperation from fellow researchers; however notice the substantial disparity.

In a related survey, geo-locating Twitter users (Jurgens et al. 2015), have demonstrated that the real world performance can be much less accurate than the lab-tested system. The performance of the 9 surveyed systems declined over time (the projected performance, i.e. the number of Twitter posts able to be geo-located, would halve every 4 months). This is another reason for conducting a comprehensive comparative study such as ours, to find out whether reported results hold during independent replication.

### Error analysis

The current geoparsers and NER taggers struggle with three main kinds of NLP challenges giving rise to a need for a deep semantics geoparser to address them. The state-of-the-art in NER is between F = 0.7 to F = 0.9 (Speck and Ngomo 2014; Ratinov and Roth 2009), depending on the type of corpus (CoNLL gets the highest scores while MUC is more challenging). Twitter NER is the most challenging due to its informal nature (Li et al. 2012). Solving the following problems is going to involve solving some common NER challenges as recognising locations in plain text is a specialised form of NER.

#### What to do with metonymy?

Perhaps the greatest weakness of current geoparsing technology is the lack of understanding of metonymy on the parsers’ side. That is, relying too much on syntactic pattern matching and the dominant sense of the entity in question at the expense of taking important cues from the surrounding words. Understanding metonymy means that the geoparser should be able to predict whether a word at the centre of some context is likely to be a location or not without knowing what the word is. Let’s illustrate with a few examples or common context errors.

In a sentence “I think I may be suffering from *Stockholm* syndrome.”, Stockholm will likely be tagged as a location because syntactically it matches a place name regardless of the clear semantic context that indicates otherwise. In “I worked with *Milan/Paris/Clinton* for 5 years.” the same is true in spite of a human clearly labelling the entity as a personal name. This is a typical example of a lack of understanding of metonymy. “*London* voted to stay in the EU.” London denotes the people in this case, not the place.

There are more subtle examples such as “She studies *at* Edinburgh.” (ORG) versus “She studies *in* Edinburgh.” (LOC) A human annotator would be able to discern Edinburgh as an institution and location given the context. A geoparser has to demonstrate the ability to understand the semantics of the context to avoid making and propagating mistakes to the geocoding stage.

#### Entity boundary errors

An example of this is “We had an amazing time at *Six Flags Over Texas* (name of an amusement park).” where only Texas is incorrectly matched as a one-word entity by all five featured geoparsers. The penalty is a huge geocoding imprecision of this toponym. Similarly, failing to recognise “*London* Road” as a whole entity, the parser commits an error if only “London” is tagged. “London Road” is almost certainly not in London so geoparsers need to more accurately recognise the boundary of the entity. However, providing that capitalisation is correct, this is an infrequent occurrence.

#### Case sensitivity

This is particularly impactful when dealing with unstructured domains like social media. When evaluated on LGL, Edinburgh and CLAVIN (using Apache OpenNLP) didn’t resolve any entities when capitalisation was removed, Topocluster (using Stanford NER) suffered a 40% dip in F-Score. For Yahoo! Placespotter, the F-Score decreased by 0.06 (10%), other metrics were unchanged.

The only outstanding geoparser is GeoTxt (using three NER taggers), which performed robustly without capitalisation. Too many geoparsers and NER taggers are currently relying on correct capitalisation to capture the full and correct entity. For inherently informal domains such as some social media, this issue will become problematic.

#### Further examples of common errors

Sentence: “$\ldots$ water main blew on the corner of *Lakeview* and *Harrison* streets, $\ldots$ ” Errors: Too greedy i.e finding and resolving “Lakeview” and “Harrison” as incorrect places (semantics and word boundary error) Correct: Either tagging nothing or for advanced parsers, find and resolve “Lakeview Street” and “Harrison Street” in the wider context of “Pineville, LA”.

Sentence: “Twin sisters Lindsay and *Charlotte* Graham, students $\ldots$” Errors: Despite clear context, “Charlotte” is tagged as a city by all but one system (semantics error) Correct: No entities found here. Geoparser greed is a common theme costing precision, but may serve to increase recall.

Sentence: “A *Rolling Meadows* resident used his car $\ldots$” Errors: All but one geoparser missed this location despite obvious context (semantics error). Correct: Tag and resolve “Rolling Meadows”, seems obvious but this is another theme of missing entities despite clear context (such as “*Mansfield* council to vote today $\ldots$”—all but one geoparser missed this mention, but not other mentions of “Mansfield” in the same paragraph).

Sentence: “The *Athens* Police Department is asking $\ldots$ ” Errors: Geocoding imprecision, confusing Texas with Greece despite clues in context (Henderson County) Correct: Athens, Texas—the clue is in the next sentence. The same mistake is made with *Berlin, Connecticut* in the context of “*Hartford*, $\ldots$ *Middletown*” and “Meeting in *Alexandria* will discuss $\ldots$ ” in the context of “Minnesota”. This particular error theme shows that the geoparsers don’t yet leverage the information generously provided in the context for a more careful and deliberate assignment of coordinates.

This use case is an illustration of incorrect labelling by the NER parsers. Several geoparsers in this paper use Stanford, Apache NLP, Illinois and/or Gate NER to perform geotagging. Example sentences: “*Bala* is a market town and community in $\ldots$” or “*Hamilton* is the seat and most populous city $\ldots$” or “*Santa Cruz* is the county seat and largest city $\ldots$ ” Errors: Incorrect label (Person) assigned by most parsers. This is despite the ample and unambiguous context. Correct: Observe the strong cues in context and assign location label.

## Conclusion

In our detailed analysis, we examined and presented the state-of-the-art systems in the geographical parsing domain. The comparative replication results showed that although useful in providing an additional dimension to the data, the systems still have several limitations. To that end, the current level of output integrity of the featured systems mainly allows for this technology to be used as supplementary input with acknowledgment of its limits rather than a high fidelity, geographical data output stream suitable for further processing “as is”. This conclusion does not mean to reflect negatively on the implementation of the software, rather it serves to show the complexity of the task of coordinating the identification of location entities and their accurate disambiguation using the contextual cues in establishing the coordinates.

The new generation of geoparsers needs to employ the understanding of the meaning of context beyond syntactical and word form. It then has to use the cues extracted to intelligently and consistently query the integrated geographical database. An example of that is correctly processing a phrase such as “They are *40 miles west of the Statue of Liberty* $\ldots$”. To detect and resolve these toponyms requires a more sophisticated AI parsing capability and an additional database of place coordinates. This is the next frontier for the next generation of geoparsers. The challenges of geoparsing make this task a worthwhile problem to solve as the benefits of mass scale text document (geographical) annotation can range from generating high quality social sciences and humanities research to accurately monitoring the world’s media for geographical events to facilitating the communication between robots and people. The possibilities for this technology are limitless.

## Footnotes

1
https://en.wikipedia.org/wiki/Wikipedia:WikiProject_Geographical_coordinates.

2
http://unstats.un.org/unsd/geoinfo/UNGEGN/.

3
https://opennlp.apache.org/.

4
http://nlp.stanford.edu/ner/.

5
https://pleiades.stoa.org/.

6
http://demo2-opener.rhcloud.com/welcome.action.

7
http://www.opencalais.com/opencalais-demo/.

8
https://gate.d5.mpi-inf.mpg.de/webaida/.

9
https://clavin.bericotechnologies.com/about-clavin/.

10
https://opennlp.apache.org/.

11
http://www.geonames.org/.

12
https://clavin.bericotechnologies.com/clavin-core/index.html.

13
https://developer.yahoo.com/boss/geo/docs/key-concepts.html.

14
http://edina.ac.uk/unlock/.

15
https://www.ltg.ed.ac.uk/software/geoparser/.

16
http://www.naturalearthdata.com/.

17
http://www.geotxt.org/api/.

18
http://nlp.stanford.edu/software/CRF-NER.shtml.

19
https://gate.ac.uk/sale/tao/splitch6.html#chap:annie.

20
http://cogcomp.cs.illinois.edu/page/demo_view/ner.

21
https://github.com/utcompling/WarOfTheRebellion.

22
https://catalog.ldc.upenn.edu/LDC2011T02.

23
https://github.com/milangritta/WhatsMissingInGeoparsing.

24
http://www.geonames.org/export/.

25
http://www.geonames.org/wikipedia/.

26
https://www.mediawiki.org/wiki/API:Main_page.

27
https://pypi.python.org/pypi/wikipedia.

28
http://www.geonames.org/export/wikipedia-webservice.html#wikipediaSearch.

29
www.geonames.org/wikipedia/wikipedia_features.html.

30
https://github.com/milangritta/WhatsMissingInGeoparsing.

31
https://en.wikipedia.org/wiki/Wikipedia:Manual_of_Style/Lead_section.

32
https://pypi.python.org/pypi/wikipedia.

33
http://www.geonames.org/products/premium-data.html.
